# Syphilis among adult males with a history of male-to-male sexual contact living with diagnosed HIV in New York State (excluding New York City): The challenge of intersecting epidemics

**DOI:** 10.1371/journal.pone.0226614

**Published:** 2019-12-18

**Authors:** Rachel Hart-Malloy, Mark Rosenthal, Wendy Patterson, Salvatore Currenti, Travis O’Donnell, Jayleen KL Gunn

**Affiliations:** 1 Division of HIV/STD Epidemiology, Evaluation, and Partner Services, AIDS Institute, New York State Department of Health, Albany, New York, United States of America; 2 Department of Epidemiology and Biostatistics, University at Albany, State University of New York, Rensselaer, New York, United States of America; 3 United States Public Health Service, Atlanta, Georgia, United States of America; Agencia de Salut Publica de Barcelona, SPAIN

## Abstract

Since 2009, syphilis has been increasing in New York State (NYS) excluding New York City (NYC) among men with a history of male-to-male sexual contact (MSM). Because MSM make up a disproportionate number of new HIV infections, this study aims to: 1) establish yearly rates of early syphilis diagnosis, 2) assess factors associated with early syphilis diagnosis, and 3) describe missed opportunities for earlier diagnosis of syphilis among MSM living with diagnosed HIV(MSMLWDH) in NYS, excluding NYC. A cohort of adult MSMLWDH alive in 2013 were followed through 2016 to identify individuals with at least one early syphilis diagnosis between July 2014 and December 2016. Early syphilis diagnosis rates were calculated for 2015 and 2016. Crude relative risks and 95% confidence intervals were calculated to determine associations between available covariates and both syphilis diagnosis and missed opportunities. Missed opportunities were defined as reports of an HIV-related laboratory test within a given window corresponding to syphilis staging where syphilis testing was not performed at the same time. Of 7,512 MSMLWDH, 50.0% were non-Hispanic white, 85.4% aged ≥35, and 320(4.3%) had an early syphilis diagnosis. Yearly rates were: 1,838/100,000, and 1,681/100,000 in 2015 and 2016, respectively. Persons who were non-Hispanic black, living with diagnosed HIV for less than three years, aged <45, and were always virally suppressed or always in HIV care were significantly more likely to have a syphilis diagnosis. Over half of individuals had evidence of a missed opportunity for earlier syphilis diagnosis. Syphilis stage at diagnosis, older age, and syphilis diagnosis not concurrent with an HIV-related laboratory test were associated with a higher likelihood of having a missed opportunity. This study supports high interrelatedness of the syphilis and HIV epidemics among MSM. Since syphilis can impact HIV viral load suppression status, efforts to end the HIV epidemic need to be coupled with syphilis elimination efforts.

## Introduction

The rate of diagnosis of HIV infection has been declining in the United States, falling 10% between 2012 and 2016.[1] Most people newly diagnosed with HIV are men who report a history of male-to-male sexual contact (MSM), who have an estimated 1 in 6 lifetime risk of acquiring HIV.[1, 2] Similarly, MSM continue to experience the highest estimated prevalence of HIV compared to people with other reported transmission categories.[1, 3] Black and Hispanic MSM are particularly disproportionately impacted, with 1 in 2 black MSM, and 1 in 5 Hispanic/Latino MSM having an estimated lifetime risk of acquiring HIV compared to 1 in 11 white MSM.[2] In New York State (NYS), excluding New York City (NYC), there are more people diagnosed with HIV who indicated a history of MSM than all other HIV risk groups combined and rates of new HIV diagnoses among non-Hispanic black and Hispanic individuals were 7.2 and 5.4 times higher, respectively, than the rate for non-Hispanic white individuals.[4]

Rates of primary and secondary syphilis, the most infectious stages of syphilis, have been increasing in the United States almost every year since reaching a historic low in 2000, and increased 10.5% from 2016 to 2017 alone.[5, 6] While rates are increasing among both males and females, and across all racial/ethnic groups, males accounted for 90% of all primary and secondary syphilis diagnoses in 2017, and rates among non-Hispanic black individuals were 4.5 times higher than non-Hispanic white individuals, mirroring the disparities observed for HIV.[5] Roughly 80% of males whose case surveillance record included information on the sex of sex partners reported having intercourse with males.[5] Estimated primary and secondary syphilis rates among MSM greatly exceed those of men who report having sex with women only (309.0 per 100,000 compared to 2.9 per 100,000 in 2015).[7, 8] Non-Hispanic black MSM are also disproportionately impacted with respect to primary and secondary syphilis (604.3 per 100,000 compared to 170.0 per 100,000 among non-Hispanic white MSM).[7, 8] The number of early latent syphilis diagnoses, defined as a latent stage of syphilis where the infection occurred within the past year, has also reached new highs with a 17.6% increase from 2016–2017.[5] NYS, excluding NYC, is seeing comparable statistics with a 4.9% increase of primary and secondary syphilis from 2016 to 2017, 90.9% of primary and secondary diagnoses in 2017 among males, and rates among non-Hispanic black individuals six times higher than rates among non-Hispanic white individuals.[9] Early latent syphilis diagnoses increased 16.8% from 2016 to 2017 in NYS, excluding NYC.[9]

The links between syphilis and HIV are well established from both clinical and epidemiologic perspectives. Multiple studies have shown that syphilis infection, and/or co-infection with other sexually transmitted infections (STIs) (i.e. herpes, chancroid) can facilitate HIV acquisition and transmission.[10–17] Among MSM, syphilis infection has been shown to increase the risk of HIV acquisition between 2 and 3 fold.[18] One study found that 1 in 20 MSM diagnosed with primary or secondary syphilis was subsequently diagnosed with HIV within a year of their syphilis diagnosis.[19] Rates of incident syphilis have also been shown to be high among MSM living with diagnosed HIV (MSMLWDH).[20–22] Specifically among persons living with diagnosed HIV (PLWDH) syphilis infection may increase HIV viral load and decrease CD4 count, though study findings suggest antiretroviral therapy (ART) adherence may mitigate these effects.[23–27] For these reasons, medical guidelines and recommendations call for screening PLWDH for syphilis, with more frequent screening recommended for those at highest risk of syphilis infection.[28–33]

Despite screening recommendations, some evidence suggests there are opportunities for improving the percentage of PLWDH who receive syphilis screening. According to data from the United States-based Medical Monitoring Project, only 71% of sexually active MSMLWDH had evidence of being tested for syphilis in the past year.[15] In NYS excluding NYC, only about 60% of PLWDH ≥19 years of age enrolled in Medicaid were screened for syphilis in the past year.[34]

Early syphilis, defined as a syphilis infection less than one-year in duration, includes primary, secondary, and early latent syphilis diagnoses. Roughly 30% of early syphilis diagnoses reported annually in NYS excluding NYC are among PLWDH; among males in NYS diagnosed with early syphilis, over 80% report having sex with males.[9, 35] Increases in syphilis, if unaddressed, have the potential to undermine efforts to end the HIV epidemic. Since a significant percentage of newly diagnosed early syphilis cases are among PLWDH, this group represents a priority population with respect to syphilis testing, treatment, and prevention of onward transmission.

In NYS excluding NYC, the degree to which early syphilis is being diagnosed among MSMLWDH and the timeliness of that diagnosis are not well understood. Therefore, the aims of this study were to: 1) establish the yearly rate of early syphilis diagnosis in the cohort, 2) assess factors associated with early syphilis diagnosis, and 3) describe missed opportunities for earlier detection of syphilis among those diagnosed with syphilis among MSMLWDH in NYS, excluding NYC.

## Methods

### Study population

Persons eligible for inclusion were males meeting the Centers for Disease Control and Prevention (CDC) HIV case definition as of January 1, 2013, who were over the age of 18 years as of January 1, 2014, with a reported history of male-to-male sexual contact, whose residential address was never documented to be outside of NYS excluding NYC from 2013 through 2016, and whose vital status indicated they were alive at the end of 2016. Males were categorized as MSM if they had ever reported MSM behavior prior to HIV acquisition.[36] All HIV-related data were extracted from NYS and NYC health departments’ enhanced HIV/AIDS Reporting Systems (eHARS), which includes all HIV-related laboratory test results for persons living or receiving HIV care in NYS. Because NYS and NYC eHARS are not linked, information was extracted from both eHARS to capture all available information. In accordance with NYS Public Health Law, clinicians must report all new or previously unreported diagnoses of HIV infection, AIDS or HIV illness within 14 days of diagnosis to the NYS Department of Health (NYSDOH).[37, 38]

Employing a retrospective cohort study design, this cohort of individuals living with diagnosed HIV was matched to early syphilis diagnoses reported to the NYS Communicable Disease Electronic Surveillance System (CDESS) between July 1, 2014 to December 31, 2016. The study follow-up time was selected to increase the probability that the syphilis infection, regardless of when it was reported to CDESS, occurred after an individual’s HIV diagnosis; individuals included in the study had to be diagnosed with HIV for at least one year prior to the start of the July 1, 2014 follow-up. Early syphilis diagnoses were defined as diagnoses meeting CDC case definitions for primary, secondary, or early latent syphilis, and were staged per CDC case definition at the time of the diagnosis (reflecting the results of laboratory data, physical examination, and partner services investigation information).[39] Persons from the cohort who matched to at least one early syphilis diagnosis during the follow-up period were defined as having had a subsequent early syphilis diagnosis. For persons with multiple syphilis diagnoses, only the first diagnosis was included.

### Statistical analysis

#### Yearly diagnosis rates of early syphilis among cohort of adult MSMLWDH

Yearly unadjusted rates of early syphilis diagnosis per 100,000 MSMLWDH in NYS excluding NYC were calculated for calendar years in which full years of data were available. Yearly rates were calculated for 2015 and 2016 by dividing the number of early syphilis diagnoses each year by the study population and presented as a rate per 100,000 persons per year; for the 2016 rate, diagnoses in 2015 were removed from the study population. Rates for 2014 were not calculated because early syphilis diagnoses were only matched for the second half of the year.

#### Associations with diagnosis of early syphilis among cohort of adult MSMLWDH

Bivariate and multivariable analyses were conducted to determine associations between available covariates and subsequent early syphilis diagnosis. For the bivariate analysis, unadjusted risk ratios (RRs) and 95% confidence intervals (CIs) were calculated for all individual- and zip-code-level covariates. Only covariates significantly associated with the outcome in the bivariate analyses were included in the multivariable analysis. The final multivariable model was restricted to covariates that remained significant at a significance level of <0.05 and were not highly collinear. Collinearity was assessed by calculating the variance inflation factor (VIF) for all variables considered for inclusion in the multivariable model. Available covariates included: race/ethnicity, age, duration of HIV positive status, HIV care status, HIV viral load suppression status, and socioeconomic variables. All demographic and HIV-related outcome variables were extracted from eHARS. Race/ethnicity was categorized as non-Hispanic white, non-Hispanic black, Hispanic, or other. Age was defined as one’s age at the start of the study period and was categorized into 10-year increments after the age of 24; age groups were defined as follows: 18–24, 25–34, 35–44, and 45+. Ages higher than 45 were collapsed to one category to ensure adequate cell sizes in the analysis.

Duration of HIV positive status was calculated as the difference in years from the initial HIV diagnosis until the study start year of 2013. Presence of a CD4, HIV viral load, or HIV genotype test result reported to the NYSDOH was used as a proxy for HIV care. HIV care status from January 1, 2013 to December 31, 2016 was defined as: 1) always in care: presence of at least one HIV-related laboratory test per year of follow-up (i.e. HIV-related laboratory testing collection dates ≤ 13 months apart); 2) sometimes in care: at least one HIV-related laboratory test during follow-up [i.e. HIV-related laboratory testing collection date(s) >13 months]; and 3) never in care: no evidence of an HIV-related laboratory test reported to the NYSDOH within the study period. HIV care data prior to the start of follow-up was included to allow individuals time to get into HIV care.

HIV viral load suppression status was defined using quantitative viral load results reported from January 1, 2013 to December 31, 2016, and was categorized as follows: 1) always suppressed: all viral loads reported during follow-up were <200 copies/mL, and the HIV viral load laboratory test collection dates were ≤13 months apart, 2) sometimes suppressed: all viral load test results reported during follow-up were <200 copies /mL but gaps of HIV care >13 months between tests, or any viral load test reported during follow-up was ≥200 copies /mL, but there was at least one viral load test <200 copies/mL, and 3) never suppressed: no reported viral load tests during follow-up were <200 copies/mL, or there were no reported viral load test dates reported during follow-up.

Zip-code level socioeconomic factors included were: percent below poverty, percent unemployed, and percent uninsured. These values were assigned to individuals based on their zip code of residence in 2016. Socioeconomic measures were extracted from the American Community Survey’s 2016 five-year estimates and were categorized into quartiles.[40] The 2016 zip code was used to allow for a more complete match to the American Community Survey.

#### Missed opportunities for syphilis diagnosis

People who had both evidence of any HIV-related laboratory testing during the study period and a subsequent syphilis diagnosis were assessed for missed opportunities for earlier syphilis diagnosis. Missed opportunities were defined by the absence of a syphilis diagnosis on the same date as an HIV care visit, defined as an HIV-related laboratory test (i.e. CD4, HIV viral load, or HIV genotype test), within a given window corresponding to syphilis staging as follows: for individuals diagnosed with secondary syphilis, any HIV-related laboratory test from one week to three months prior to the secondary syphilis diagnosis was defined as a missed opportunity for earlier syphilis detection.[39, 41] For those diagnosed with early latent syphilis, HIV-related laboratory tests from one week to six months prior to the syphilis diagnosis were defined as a missed opportunity.[39] These time-bound cut points were chosen based on the natural course of syphilis infection.[42] The presence or absence of a missed opportunity was determined for each person based on their syphilis stage at diagnosis. Individuals diagnosed with primary syphilis, or those with no reported HIV care dates, were excluded from the potential missed opportunity analysis.

Bivariate and multivariable associations between the following covariates and having a missed opportunity were assessed: syphilis disease stage, race/ethnicity, age, duration of HIV positive status, and syphilis diagnosis on same day as HIV care. HIV care and viral load suppression status were not included in this analysis as covariates given that persons eligible for this analysis were selected based upon receipt of HIV-related laboratory tests and would therefore introduce bias in measuring these covariates. Multivariable modeling methods for this analysis mirrored those employed for examining the relationship between the listed covariates and a subsequent early syphilis diagnosis.

All analyses were performed using SAS version 9.4 (SAS Institute, Cary, NC). RRs were calculated using “proc genmod.” This secondary data analysis was considered exempt from human subject’s review by the NYSDOH Institutional Review Board.

## Results

### Study population

There were 7,512 MSMLWDH aged ≥18 years living in NYS from 2013–2016 included in this cohort. Most individuals in this cohort were non-Hispanic white (3,755; 50.0%), ≥35 years of age (6,419; 85.5%) and had been diagnosed with HIV for 7 or more years (5,381; 71.6%) (Table 1). Less than half (3,302; 44.0%) of MSMLWDH in this cohort were always virally suppressed, and two-thirds (4,982; 66.3%) had at least one HIV care date every 13 months. Over half (4,339; 57.8%) of MSMLWDH lived in a zip code where more than 10.2% of people live below poverty. MSMLDWH residing in a zip code with an unemployment rate higher than 9.2% made up 22.1% of the cohort, and 1,907 (25.4%) lived in zip code where more than 9.3% of the population was uninsured. During the study period, 320 (4.3%) MSMLWDH had an early syphilis diagnosis.

**Table 1:** Descriptive analysis for cohort of adult males with a history of male-to-male sexual contact living with diagnosed HIV, and syphilis diagnosis status: New York state excluding New York city, 2013–2016.

	Total		Syphilis Diagnosis		No Syphilis		Unadjusted Risk Ratio and 95% CL^1^	Adjusted Risk Ratio and 95% CL^1^
	(n)	col %	(n)	col %	(n)	col %		
Total	7,512	-	320	-	7,192	-	-	
**Race/Ethnicity**								
Non-Hispanic White	3,755	50.0%	122	38.1%	3,633	50.5%	*Ref*.	*Ref*.
Non-Hispanic Black	1,386	18.5%	88	27.5%	1,298	18.0%	**1.95 (1.49–2.55)**	**1.44 (1.10–1.89)**
Hispanic	1,434	19.1%	66	20.6%	1,368	19.0%	**1.41 (1.05–1.89)**	1.03 (0.77–1.39)
Other^2^	937	12.5%	44	13.8%	893	12.4%	**1.44 (1.03–2.02)**	1.19 (0.85–1.66)
**Age Group**^**3**^								
18–24	137	1.8%	17	5.3%	120	1.7%	**5.74 (3.55–9.30)**	**2.99 (1.77–5.05)**
25–34	956	12.7%	110	34.4%	846	11.8%	**5.33 (4.13–6.87)**	**2.94 (2.18–3.95)**
35–44	1,281	17.1%	82	25.6%	1,199	16.7%	**2.96 (2.24–3.92)**	**2.12 (1.59–2.84)**
45+	5,138	68.4%	111	34.7%	5,027	69.9%	*Ref*.	*Ref*.
**Viral Load** **≤200 c/ml**								
Always^4^	3,302	44.0%	132	41.3%	3,170	44.1%	**2.44 (1.51–3.92)**	**3.36 (2.09–5.42)**
Sometimes^5^	3,052	40.6%	169	52.8%	2,883	40.1%	**3.37 (2.11–5.40)**	**3.60 (2.26–5.74)**
Never^6^	1,158	15.4%	19	5.9%	1,139	15.8%	*Ref*.	*Ref*.
**HIV Care Status**								
Always^7^	4,982	66.3%	225	70.3%	4,757	66.1%	**5.18 (2.57–10.40)**	-
Sometimes^8^	1,611	21.4%	87	27.2%	1,524	21.2%	**6.20 (3.02–12.70)**	-
Never^9^	919	12.2%	8	2.5%	911	12.7%	*Ref*.	-
**Duration of HIV Positive Status**								
1–3 Years	1,098	14.6%	100	31.3%	998	13.9%	**7.83 (5.38–11.4)**	**3.81 (2.50–5.81)**
4–6 Years	1,033	13.8%	81	25.3%	952	13.2%	**6.74 (1.48–9.92)**	**3.89 (2.56–5.91)**
7–13 years	2,287	30.4%	103	32.2%	2,184	30.4%	**3.87 (2.66–5.64)**	**2.90 (1.97–4.27)**
Greater than 13 years	3,094	41.2%	36	11.3%	3,058	42.5%	*Ref*.	*Ref*.
**Zip Code Percent Below Poverty**								
0% - 5.7%	1,100	14.6%	56	17.5%	1,044	14.5%	1.17 (0.86–1.59)	-
5.7% - 10.2%	1,824	24.3%	69	21.6%	1,755	24.4%	0.86 (0.63–1.17)	-
10.2% - 16.4%	1,445	19.2%	62	19.4%	1,383	19.2%	0.96 (0.68–1.34)	-
16.4% and higher	2,894	38.5%	129	40.3%	2,765	38.4%	*Ref*.	-
Missing	249	3.3%	4	1.3%	245	3.4%	-	-
**Zip Code Unemployment Rate**								
Below 4.4%	763	10.2%	35	10.9%	728	10.1%	0.95 (0.54–1.67)	-
Between 4.4% and 6.5%	2,355	31.3%	82	25.6%	2,273	31.6%	0.93 (0.65–1.32)	-
Between 6.5% and 9.2%	2,481	33.0%	128	40.0%	2,353	32.7%	1.09 (0.84–1.42)	-
Higher than 9.2%	1,663	22.1%	70	21.9%	1,593	22.1%	*Ref*.	-
Missing	250	3.3%	5	1.6%	245	3.4%	-	-
**Zip Code Percent Uninsured**								
Below 4.0%	1,006	13.4%	48	15.0%	958	13.3%	1.05 (0.73–1.49)	-
Between 4.0% and 6.3%	1,893	25.2%	62	19.4%	1,831	25.5%	0.72 (0.52–1.01)	-
Between 6.3% and 9.3%	2,458	32.7%	118	36.9%	2,340	32.5%	1.04 (0.77–1.39)	-
Above 9.3%	1,907	25.4%	88	27.5%	1,819	25.3%	*Ref*.	-
Missing	248	3.3%	4	1.3%	244	3.4%	-	-

1. Bolded results indicate *p*-value<0.05

2. Other race includes Asian/Pacific Islander, Native American, Multi-race, and unknown

3. Persons age as of January 1, 2014

4. Always: from January 1, 2013 to December 31, 2016, all viral loads must be <200 viral copies/mL and HIV viral load laboratory test collection dates needed to be < 13 months apart

5. Sometimes includes one of the following two conditions: 1) all test results were <200 copies/mL but there were gaps of HIV care > 13 months between tests or 2) or any test was ≥ 200 copies/mL, but there was at least one test < 200 copies/mL.

6. Never: zero tests <200 copies/mL or no viral load test dates reported to the NYSDOH

7. Always: presence of at least one HIV-related laboratory test per year of follow-up (i.e. HIV-related laboratory testing collection dates < 13 months apart)

8. Sometimes: at least one HIV-related laboratory test during follow-up (i.e. HIV-related laboratory testing collection date(s) >13 months)

9. Never: no evidence of an HIV-related laboratory reported to the NYSDOH within study period

#### Yearly diagnosis rates of early syphilis among cohort of adult MSMLWDH

For the years in which diagnosis rates were calculated, there were 137 early syphilis diagnoses in 2015, and 123 in 2016. The yearly rates of early syphilis diagnosis were 1,838 per 100,000 MSMLWDH and 1,681 per 100,000 MSMLWDH in 2015 and 2016, respectively.

#### Associations with diagnosis of early syphilis among cohort of adult MSMLWDH

Race/ethnicity, age, viral load suppression status, HIV care status, and duration of HIV infection were all significantly associated with a diagnosis of early syphilis (Table 1). Non-Hispanic black MSMLWDH were 1.95 (95% CI: 1.49–2.55) times more likely to have an early syphilis diagnosis than non-Hispanic white MSMLWDH. Age was inversely associated with early syphilis diagnosis; persons 18–24 years of age were 5.74 (95% CI: 3.55–9.30) times more likely to be diagnosed with early syphilis than those aged 45 and older. MSMLWDH who were categorized as always virally suppressed or always in HIV care during the study period were more likely to have an early syphilis diagnosis [RR: 2.44 (95% CI:1.51–3.92) and RR 5.18 (95% CI:2.57–10.40), respectively]. MSMLWDH for less than three years were 7.83 (95% CI: 5.38–11.40) times more likely to be diagnosed with early syphilis than those who had been living with HIV for greater than 13 years. Zip code level percent below poverty, percent unemployment, and percent uninsured were not significantly associated with early syphilis diagnosis among MSMLWDH.

The final multivariable model included race/ethnicity, age, viral load suppression status, and duration of HIV infection. HIV care status and viral load suppression status were collinear (the VIF was 3.73 and 3.78 for HIV care status and viral load suppression status, respectively); therefore, only viral load suppression status was retained for the final analysis. After adjusting for all other variables in the model, non-Hispanic black MSMLWDH were 1.44 (95% CI: 1.10–1.89) times more likely to have an early syphilis diagnosis than non-Hispanic white MSMLWDH (Table 1). Those aged 18–24 were 2.99 (95% CI: 1.77–5.05) times more likely to have an early syphilis diagnosis than those aged 45 or older. MSMLWDH who were categorized as always virally suppressed during the study period were more likely to have an early syphilis diagnosis [adjusted risk ratio (aRR): 3.36 (95% CI:2.09–5.42)] than those who were never suppressed. Those living with HIV for less than three years were 3.81 (95% CI: 2.50–5.81) times as likely to have a syphilis diagnosis than those living with HIV for more than 13 years.

#### Missed opportunities for syphilis diagnosis

Of the 320 MSMLWDH with an early syphilis diagnosis, 261 were analyzed for missed opportunities for syphilis diagnosis during an HIV care visit (excludes eight individuals with no HIV-related laboratory tests and 51 individuals diagnosed with primary syphilis). Of individuals included in the analysis, 145 (55.6%) had at least one missed opportunity for syphilis diagnosis during an HIV care visit (Table 2). MSMLWDH diagnosed with early latent syphilis were 1.96 (95% CI: 1.48–2.58) times more likely to have a missed opportunity than those diagnosed with secondary syphilis. Individuals whose syphilis diagnosis date differed from their HIV care date were 1.28 (95% CI: 1.04–1.59) times more likely to have a missed opportunity than those whose diagnosis occurred on the same date as their HIV care. Race/ethnicity, age, and duration of HIV positive status were not significantly associated with having a missed opportunity.

**Table 2:** Missed opportunities for earlier syphilis diagnosis cohort of adult males with a history of male-to-male sexual contact living with diagnosed HIV: New York state excluding New York CIty, 2013–2016.

	Total^1^		Missed Opportunity^2^				Unadjusted Risk Ratio and 95% CL^3^	Adjusted Risk Ratio and 95% CL^3^
			Yes		No			
	(n)	col %	(n)	col %	(n)	col %		
**Total**	261		145	-	116	-		
**Syphilis Disease Stage**								
Secondary Syphilis	107	41.0%	38	26.2%	69	59.5%	*Ref*.	*Ref*.
Early Latent Syphilis	154	59.0%	107	73.8%	47	40.5%	**1.96 (1.48–2.58)**	**1.97 (1.50–2.59)**
**Race/Ethnicity**^**4**^		** **						
Non-Hispanic White	106	40.6%	59	40.7%	47	40.5%	*Ref*.	-
Non-Hispanic Black	66	25.3%	34	23.4%	32	27.6%	0.93 (0.69–1.24)	-
Hispanic	52	19.9%	26	17.9%	26	22.4%	0.90 (0.65–1.24)	-
Other	37	14.2%	26	17.9%	11	9.5%	1.26 (0.96–1.65)	-
**Age Group**^**5**^								
18–24	13	5.0%	5	3.4%	8	6.9%	0.63 (0.31–1.28)	-
25–34	88	33.7%	52	35.9%	36	31.0%	0.97 (0.76–1.23)	-
35–44	70	26.8%	33	22.8%	37	31.9%	0.77 (0.57–1.04)	-
45+	90	34.5%	55	37.9%	35	30.2%	*Ref*.	-
**Duration of HIV Positive Status**								
1–3 Years	79	30.3%	46	31.7%	33	28.4%	0.97 (0.69–1.37)	-
4–6 Years	65	24.9%	38	26.2%	27	23.3%	0.97 (0.68–1.39)	-
7–13 years	87	33.3%	43	29.7%	44	37.9%	0.82 (0.57–1.18)	-
Greater than 13 years	30	11.5%	18	12.4%	12	10.3%	*Ref*.	-
**Same Day Syphilis Diagnosis**^**6**^								
Yes	165	63.2%	83	57.2%	82	70.7%	*Ref*.	*Ref*.
No	96	36.8%	62	42.8%	34	29.3%	**1.28 (1.04–1.59)**	**1.30 (1.09–1.57)**

1. Excludes those who had no HIV-related laboratory test collection dates or were diagnosed with primary syphilis (N = 59)

2. Missed opportunity: an HIV care date within 3 months prior to a secondary syphilis diagnosis, or 6 months prior to an early latent syphilis diagnosis

3. Bolded results indicate *p*-value<0.05

4. Other race includes Asian/Pacific Islander, Native American, Multi-race, and unknown

5. Persons age as of January 1, 2014

6. Same day syphilis diagnosis: syphilis diagnosed date matches an HIV care date

The final multivariable model included disease stage at diagnosis and whether the syphilis diagnosis occurred on the same day as an HIV care visit. After adjusting for other variables, those diagnosed with early latent syphilis were 1.97 (95% CI: 1.50–2.59) times more likely to have a missed opportunity than those diagnosed with secondary syphilis (Table 2). Those who were not diagnosed with syphilis on the same day as their HIV care were 1.30 (95% CI: 1.09–1.57) times as likely to have a missed opportunity compared to those who were diagnosed concurrently with their HIV care.

#### Post hoc analysis

As a post hoc analysis, early syphilis diagnoses that occurred on the same date as an HIV care visit (defined as presence of an HIV-related laboratory test result) were quantified for each person to assess the degree to which syphilis is diagnosed at the same time as one’s HIV care (Table 3). Excluding eight individuals who had no HIV-related laboratory tests reported dates, there were 312 people diagnosed with early syphilis, of which 191 (61.2%) were diagnosed on the same day as the HIV care visit. Due to small cell sizes, ‘Never’ and ‘Sometimes’ virally suppressed were combined resulting in the co-variate being recategorized as ‘Yes’/’No’ to always virally suppressed. Syphilis disease stage at diagnosis was the only factor significantly associated with a same day diagnosis. MSMLWDH diagnosed with early latent syphilis were 1.41 (95% CI: 1.06–1.88) times more likely to have a same day diagnosis than those with primary syphilis. There was no significant difference between those diagnosed with secondary syphilis compared to primary syphilis. As no other variables were significantly associated with having a syphilis diagnosis on the same day as HIV care, a multivariable model was not constructed for the post-hoc analysis.

**Table 3:** Syphilis diagnosis on the same day as HIV care among MSM over the age of 18 living with HIV by demographic factors, New York state excluding New York city, 2016.

	Total^1^		Same Day Diagnosis^2^				Risk Ratio and 95% CL^3^
			Yes		No		
	(n)	col %	(n)	col %	(n)	col %	
**Total**	312		191	-	121	-	
**Syphilis Disease Stage**							
Primary Syphilis	51	16.3%	26	13.6%	25	20.7%	*Ref*.
Secondary Syphilis	107	34.3%	54	28.3%	53	43.8%	0.99 (0.71–1.37)
Early Latent Syphilis	154	49.4%	111	58.1%	43	35.5%	**1.41 (1.06–1.88)**
**Race/Ethnicity**^**4**^		** **					
Non-Hispanic White	120	38.5%	80	41.9%	40	33.1%	*Ref*.
Non-Hispanic Black	84	26.9%	45	23.6%	39	32.2%	0.80 (0.63–1.02)
Hispanic	64	20.5%	40	20.9%	24	19.8%	0.94 (0.75–1.18)
Other	44	14.1%	26	13.6%	18	14.9%	0.89 (0.67–1.17)
**Age Group**^**5**^							
18–24	17	5.4%	11	5.8%	6	5.0%	0.99 (0.68–1.45)
25–34	106	34.0%	59	30.9%	47	38.8%	0.85 (0.69–1.06)
35–44	80	25.6%	50	26.2%	30	24.8%	0.96 (0.77–1.19)
45–54	109	34.9%	71	37.2%	38	31.4%	*Ref*.
**Viral Load Always** **≤200 c/mL**							
Yes	132	42.3%	87	45.5%	45	37.2%	1.14 (0.96–1.34)
No	180	57.7%	104	54.5%	76	62.8%	*Ref*.
**HIV Care Status**							
Always^6^	225	72.1%	145	75.9%	80	66.1%	1.22 (0.98–1.52)
Sometimes in Care^7^	87	27.9%	46	24.1%	41	33.9%	*Ref*.
Never in Care	-	-	-	-	-	-	*excluded*
**Duration of HIV Positive Status**							
1–3 Years	98	31.4%	61	31.9%	37	30.6%	0.83 (0.65–1.06)
4–6 Years	79	25.3%	42	22.0%	37	30.6%	**0.71 (0.54–0.94)**
7–13 years	99	31.7%	61	31.9%	38	31.4%	0.82 (0.64–1.05)
Greater than 13 years	36	11.5%	27	14.1%	9	7.4%	*Ref*.
**Missed Opportunity**^**8**^							
Yes	145	46.5%	83	43.5%	62	51.2%	*Ref*.
No	167	53.5%	108	56.5%	59	48.8%	1.13 (0.94–1.35)

1. Excludes those who had no HIV-related laboratory test collection dates (N = 8)

2. Same day syphilis diagnosis: syphilis diagnosed date matches an HIV care date

3. Bolded results indicate *p*-value<0.05

4. Other race includes Asian/Pacific Islander, Native American, Multi-race, and unknown

5. Persons age as of January 1, 2014

6. Always: presence of at least one HIV-related laboratory test per year of follow-up (i.e. HIV-related laboratory testing collection dates <13 months apart)

7. Sometimes: at least one HIV-related laboratory test during follow-up (i.e. HIV-related laboratory testing collection date(s) >13 months)

8. Missed opportunity: an HIV care date within 3 months prior to a secondary syphilis diagnosis, or 6 months prior to an early latent syphilis diagnosis

## Discussion

Overall, the prevalence of early syphilis diagnosis in this study population was high, with 4.3% of individuals identified as having a subsequent early syphilis diagnosis. This high proportion of MSMLWDH being diagnosed with syphilis is comparable to other studies and national reports.[5, 10, 43] The DC cohort, a well-established cohort of persons living with HIV in the District of Columbia, examined incident STIs after 32.5 months of follow-up.[10] Of the MSM in their cohort, 4.1% were diagnosed with syphilis during the follow-up period.[10] Among MSM, the high interrelatedness of syphilis and HIV in NYS is a significant public health concern that highlights the importance of syphilis screening, treatment, and prevention counseling in efforts to end the HIV epidemic.

The yearly rates of early syphilis diagnosis found in this study of 1,838 per 100,000 and 1,681 per 100,000 in 2015 and 2016 respectively, greatly exceeded the 2016 rate of 21.6/100,000 early syphilis among males ≥18 years of age in NYS.[35] However, while not a direct comparison, the rate of primary and secondary syphilis calculated among MSMLWDH from 34 states in 2014 was 1,200/per 100,000 suggesting similarly elevated national rates of syphilis among this population.[20]

Persons at highest risk of subsequent early syphilis diagnosis were young (<35 years of age), those of racial/ethnic minority, and those living with diagnosed HIV for less than seven years. Again, this finding is similar to other studies, and support the current NYS HIV Clinical Guidelines to screen HIV positive MSM at highest risk of syphilis infection for syphilis every three months.[10, 28] Unlike other studies, persons most likely to be diagnosed with early syphilis were virally suppressed throughout the follow-up time, and in consistent HIV care.[23, 24] These findings suggest individuals diagnosed with syphilis were receiving HIV care or, at minimum, were adherent to HIV treatment, and represent promising evidence to support the biomedical approach of HIV viral load suppression as an HIV transmission prevention method. It should be noted that HIV care was narrowly defined as receiving HIV-related laboratory tests (i.e. HIV viral load, CD4 or genotype) that were reported to NYSDOH. Individuals may be seeing a medical provider for HIV care and not be receiving HIV-related laboratory tests which are reported to NYSDOH at each of those visits.[44] Further medical chart reviews should be conducted to identify if a syphilis test was offered during HIV care and if the patient refused testing.

Regarding missed opportunities, while over half of the individuals diagnosed with secondary or early latent syphilis were identified as having at least one missed opportunity for earlier syphilis detection, 63.2% were diagnosed with syphilis on the same day as HIV care (Table 2). Diagnosing syphilis at the time of HIV care provides both the individual and the provider with an opportunity for more comprehensive sexual health messaging and care.

Interestingly, while persons diagnosed with early latent syphilis were more likely to be diagnosed with syphilis on the same day as HIV care, they were also more likely to have a missed opportunity for earlier detection. While the latter finding is likely an artifact of individuals infected for a longer duration simply having more time to have a missed opportunity, it speaks to the importance of pairing syphilis screening with HIV-related laboratory tests, especially for persons who are not experiencing any syphilis symptoms (or whose symptoms go unobserved). Though primary and secondary syphilis are marked by symptoms (rectal chancres, for example), these will not be apparent without rectal screening, which is not part of routine HIV care. Pairing syphilis screening with every HIV CD4 count or viral load assay has been recommended to clinicians providing care to MSM.[33] Lending further support to paired testing is that only half of those individuals diagnosed with primary or secondary syphilis in this cohort were diagnosed on the same day as HIV care. Lastly, there were no other associations found between the covariates assessed and having a missed opportunity. Therefore, while some populations are at a higher risk of a syphilis diagnosis, screening for syphilis among PLWDH should be universal.

It should be noted that the missed opportunities should be interpreted with caution given how a missed opportunity was defined. For example, physicians may decide to monitor a patient who started ART more frequently to ensure that they become virally suppressed. The expectation to screen for syphilis at such monitoring events may not be reasonable. Alternatively, one of the HIV-related laboratory tests categorized as a missed opportunity could have been the impetus for syphilis screening. For example, for persons previously virally suppressed and on ARTs, an unexplained increase in HIV viral load may have been an indication for the clinician to screen for STIs. Furthermore, the time-bound cut points chosen to assess missed opportunities could have overestimated the duration of one’s infection, therefore misclassifying HIV care dates prior to infection as missed opportunities. This misclassification would result in an overestimate of missed opportunities. Therefore, the results of the analysis regarding missed opportunities should be interpreted with caution as they may be indicative of consistent HIV clinical care for this cohort.

The optimal outcome for anyone diagnosed with syphilis would be timely identification and treatment of the infection. As this study was limited to incident syphilis, an examination into adequate syphilis treatment per the 2015 CDC STD Treatment Guidelines for this cohort was examined and compared to the HIV negative males diagnosed with incident syphilis in the same geographic area, over the same study period. For both groups, 98% of males received adequate treatment for their syphilis infection, demonstrating optimal outcomes regardless of HIV positivity.[29]

There are several limitations to this study. First, it is unknown to what degree individuals in the cohort had undiagnosed syphilis infection(s). As this is an observational cohort, information on syphilis screening cannot be obtained. However, given current screening guidelines, the authors are hopeful that syphilis has been detected in the majority of individuals in this cohort.[28] If syphilis screening is not as high as current guidelines recommend, syphilis rates could be higher than the rates observed in this study. Second, only the first syphilis diagnosis per individual within the study timeframe was included in this study. Therefore, any syphilis diagnosis prior to study start, or subsequent to the individual’s initial syphilis diagnosis, was not accounted for (N = 36). Individuals with repeat syphilis infection may have a differing level of risk. Further analyses should examine repeat syphilis and/or additional STI diagnoses in this cohort. Third, the defined cohort of MSM was based on HIV transmission risk, which does not indicate current sexual risk, and also omits individuals who did not disclose sexual risk at time of HIV diagnosis. Fourth, virally suppressed was categorized as an HIV viral load <200 copies/mL rather than <1,500 copies/mL, which research indicates is the threshold of HIV-RNA needed to sexually transmit HIV.[45–47] This categorization may have resulted in fewer individuals being classified as suppressed than the less conservative measures would assume. Supplemental analyses conducted to mirror analytics presented in Tables 1 and 3 (Table A in S1 File, and Table B in S1 File) show the results did not significantly differ when increasing the viral load cutoff from <200 copies/mL to <1,500 copies/mL. Therefore, any bias introduced is thought to be minute. Additionally, residence was based on an individual’s last known address which may not reflect one’s current address. Based on previous research in NYS, it is known that in- and out-migration to the jurisdiction occurs frequently, and that an absence of an HIV-related laboratory test might not be reflective of being not in HIV care, but rather having moved to another jurisdiction or receiving care from an organization that is not subject to state reporting laws.[44, 48] This limitation would most impact the group of individuals who were without HIV-related laboratory tests for the duration of the follow-up period who were classified as never in care. However, as per NYSDOH practice, individuals diagnosed with AIDS and have had no HIV-related laboratory tests reported to the NYSDOH for five years, or individuals diagnosed with HIV (stage 1 and 2) and have had no HIV-related laboratory tests reported for eight years, are not considered to be in NYS, and are therefore were not included this analysis. Lastly, syphilis staging might be incorrect when a patient’s serologic history could not be confirmed. However, this is true for all jurisdictions and therefore would not preclude comparing the results from this analysis to other previously conducted analyses in other jurisdictions.

## Conclusions

In conclusion, this study supports the notion of high interrelatedness of syphilis and HIV, especially among MSM. Consistent and routine screening for syphilis among MSMLWDH is needed. Continual integration of syphilis testing during routine HIV care could help increase early identification of syphilis infection and help decrease transmission rates. Biomedical approaches, such as HIV viral load suppression, have increasingly become the cornerstone of efforts to end the HIV epidemic and must be leveraged in order to maximize opportunities for STI detection, especially since STIs can increase HIV viral loads. Comprehensive sexual health continues to be paramount to ensuring HIV and STI prevention. Additional studies examining the relationship between HIV viral load suppression status and syphilis diagnosis, as well as the relationship between syphilis testing and/or diagnosis should be conducted.

## Supporting information

S1 File (Table A) Supplemental Descriptive Analysis for Cohort of Adult Males with a History of Male-to-Male Sexual Contact Living with Diagnosed HIV, and Syphilis Diagnosis Status: New York State Excluding New York City, 2013–2016. (Table B) Supplemental Analysis of Syphilis Diagnosis on the Same Day as HIV Care Among MSM over the Age of 18 Living With HIV by Demographic Factors, New York State Excluding New York City, 2016.
(DOCX)

## References

[1] Centers for Disease Control and Prevention. HIV Surveillance Report, 2017. November 2018.

[2] Hess KL, Hu X, Lansky A, Mermin J, Hall HI. Lifetime risk of a diagnosis of HIV infection in the United States. Annals of epidemiology. 2017;27(4):238–43. 10.1016/j.annepidem.2017.02.003 28325538 PMC5524204

[3] Centers for Disease Control and Prevention. Estimated HIV incidence and prevalence in the United States, 2010–2015. 2018.

[4] Bureau of HIV/AIDS Epidemiology AIDS Institute New York State Department of Health. New York State HIV/AIDS Annual Surveillance Report for Cases Diagnosed through December 2016. 2017.

[5] Division of STD Prevention National Center for HIV/AIDS Viral Hepatitis STD and TB Prevention Centers for Disease Control and Prevention. Sexually Transmitted Disease Surveillance 2017. 2018.

[6] Patton ME, Su JR, Nelson R, Weinstock H, Centers for Disease C, Prevention. Primary and secondary syphilis—United States, 2005–2013. MMWR Morb Mortal Wkly Rep. 2014;63(18):402–6. 24807239 PMC5779405

[7] de Voux A, Kidd S, Grey JA, Rosenberg ES, Gift TL, Weinstock H, et al State-Specific Rates of Primary and Secondary Syphilis Among Men Who Have Sex with Men—United States, 2015. MMWR Morb Mortal Wkly Rep. 2017;66(13):349–54. 10.15585/mmwr.mm6613a1 28384130 PMC5657910

[8] Grey JA, Bernstein KT, Sullivan PS, Kidd SE, Gift TL, Hall EW, et al Rates of Primary and Secondary Syphilis Among White and Black Non-Hispanic Men Who Have Sex With Men, United States, 2014. J Acquir Immune Defic Syndr. 2017;76(3):e65–e73. 10.1097/QAI.0000000000001508 28749823 PMC5634915

[9] New York State Department of Health. STI surveillance and partner services data (unpublished data) 2018.

[10] Lucar J, Hart R, Rayeed N, Terzian A, Weintrob A, Siegel M, et al Sexually Transmitted Infections Among HIV-Infected Individuals in the District of Columbia and Estimated HIV Transmission Risk: Data From the DC Cohort. Open Forum Infect Dis. 2018;5(2):ofy017. 10.1093/ofid/ofy017 29479550 PMC5804762

[11] Kalichman SC, Pellowski J, Turner C. Prevalence of sexually transmitted co-infections in people living with HIV/AIDS: systematic review with implications for using HIV treatments for prevention. Sex Transm Infect. 2011;87(3):183–90. 10.1136/sti.2010.047514 21330572 PMC4317792

[12] Solomon MM, Mayer KH, Glidden DV, Liu AY, McMahan VM, Guanira JV, et al Syphilis predicts HIV incidence among men and transgender women who have sex with men in a preexposure prophylaxis trial. Clin Infect Dis. 2014;59(7):1020–6. 10.1093/cid/ciu450 24928295 PMC4166980

[13] Kaplan JE, Benson C, Holmes KK, Brooks JT, Pau A, Masur H, et al Guidelines for prevention and treatment of opportunistic infections in HIV-infected adults and adolescents: recommendations from CDC, the National Institutes of Health, and the HIV Medicine Association of the Infectious Diseases Society of America. MMWR Recomm Rep. 2009;58(RR-4):1–207; quiz CE1-4. . 19357635

[14] Lynn WA, Lightman S. Syphilis and HIV: a dangerous combination. Lancet Infect Dis. 2004;4(7):456–66. 10.1016/S1473-3099(04)01061-8 . 15219556

[15] de Voux A, Bernstein K, Bradley H, Kirkcaldy RD, Tie Y, Shouse RL, et al Syphilis testing among sexually active men who have sex with men and who are receiving medical care for HIV in the United States-Medical Monitoring Project, 2013–2014. Clin Infect Dis. 2018 10.1093/cid/ciy571 . 29985985 PMC6563935

[16] Mayer KH, Venkatesh KK. Interactions of HIV, other sexually transmitted diseases, and genital tract inflammation facilitating local pathogen transmission and acquisition. Am J Reprod Immunol. 2011;65(3):308–16. 10.1111/j.1600-0897.2010.00942.x 21214660 PMC3077541

[17] Ward H, Ronn M. Contribution of sexually transmitted infections to the sexual transmission of HIV. Curr Opin HIV AIDS. 2010;5(4):305–10. 10.1097/COH.0b013e32833a8844 20543605 PMC2923028

[18] An Q, Wejnert C, Bernstein K, Paz-Bailey G, Group NS. Syphilis Screening and Diagnosis Among Men Who Have Sex With Men, 2008–2014, 20 U.S. Cities. J Acquir Immune Defic Syndr. 2017;75 Suppl 3:S363–S9. 10.1097/QAI.0000000000001412 . 28604440

[19] Pathela P, Braunstein SL, Blank S, Shepard C, Schillinger JA. The high risk of an HIV diagnosis following a diagnosis of syphilis: a population-level analysis of New York City men. Clin Infect Dis. 2015;61(2):281–7. 10.1093/cid/civ289 . 25870333

[20] Grey J, Torrone, E., Kidd, S., Bernstein, K., Weinstock, H., editor Estimated Primary & Secondary Syphilis Rates in MSM by HIV Status—34 States, 2014. 25th Conference on Retroviruses and Opportunistic Infections; 2018; Boston, MA.

[21] Shilaih M, Marzel A, Braun DL, Scherrer AU, Kovari H, Young J, et al Factors associated with syphilis incidence in the HIV-infected in the era of highly active antiretrovirals. Medicine (Baltimore). 2017;96(2):e5849 10.1097/MD.0000000000005849 28079818 PMC5266180

[22] Braunstein S, Pathela, P., Kersanske, L., editor Incidence of Sexually Transmitted Diseases Among Persons With HIV New York City 2000–2010. Conference on Retroviruses and Opportunistic Infections; 2014; Boston, MA.

[23] Lang R, Read R, Krentz HB, Ramazani S, Peng M, Gratrix J, et al Increasing incidence of syphilis among patients engaged in HIV care in Alberta, Canada: a retrospective clinic-based cohort study. BMC Infect Dis. 2018;18(1):125 10.1186/s12879-018-3038-4 29534681 PMC5851255

[24] Kofoed K, Gerstoft J, Mathiesen LR, Benfield T. Syphilis and human immunodeficiency virus (HIV)-1 coinfection: influence on CD4 T-cell count, HIV-1 viral load, and treatment response. Sex Transm Dis. 2006;33(3):143–8. 10.1097/01.olq.0000187262.56820.c0 . 16505739

[25] Taylor MM, Newman DR, Schillinger JA, Lewis FM, Furness B, Braunstein S, et al Viral Loads Among HIV-Infected Persons Diagnosed With Primary and Secondary Syphilis in 4 US Cities: New York City, Philadelphia, PA, Washington, DC, and Phoenix, AZ. J Acquir Immune Defic Syndr. 2015;70(2):179–85. 10.1097/QAI.0000000000000730 . 26090756 PMC6749136

[26] Jarzebowski W, Caumes E, Dupin N, Farhi D, Lascaux AS, Piketty C, et al Effect of early syphilis infection on plasma viral load and CD4 cell count in human immunodeficiency virus-infected men: results from the FHDH-ANRS CO4 cohort. Arch Intern Med. 2012;172(16):1237–43. 10.1001/archinternmed.2012.2706 . 22826097

[27] Buchacz K, Patel P, Taylor M, Kerndt PR, Byers RH, Holmberg SD, et al Syphilis increases HIV viral load and decreases CD4 cell counts in HIV-infected patients with new syphilis infections. AIDS. 2004;18(15):2075–9. 10.1097/00002030-200410210-00012 . 15577629 PMC6763620

[28] New York State Department of Health Clinical Guidelines Program Sexually Transmitted Infections Guidelines Committee. Management of Syphilis in Patients with HIV 2018 [cited 2018]. Available from: https://www.hivguidelines.org/sti-care/syphilis/#tab_6.

[29] Workowski KA, Bolan GA, Centers for Disease C, Prevention. Sexually transmitted diseases treatment guidelines, 2015. MMWR Recomm Rep. 2015;64(RR-03):1–137. 26042815 PMC5885289

[30] Aberg JA, Gallant JE, Ghanem KG, Emmanuel P, Zingman BS, Horberg MA, et al Primary care guidelines for the management of persons infected with HIV: 2013 update by the HIV medicine association of the Infectious Diseases Society of America. Clin Infect Dis. 2014;58(1):e1–34. 10.1093/cid/cit665 . 24235263

[31] Cantor AG, Pappas M, Daeges M, Nelson HD. Screening for Syphilis: Updated Evidence Report and Systematic Review for the US Preventive Services Task Force. JAMA. 2016;315(21):2328–37. 10.1001/jama.2016.4114 . 27272584

[32] Force USPST, Bibbins-Domingo K, Grossman DC, Curry SJ, Davidson KW, Epling JW Jr., et al Screening for Syphilis Infection in Nonpregnant Adults and Adolescents: US Preventive Services Task Force Recommendation Statement. JAMA. 2016;315(21):2321–7. 10.1001/jama.2016.5824 . 27272583

[33] Zetola NM, Engelman J, Jensen TP, Klausner JD. Syphilis in the United States: an update for clinicians with an emphasis on HIV coinfection. Mayo Clin Proc. 2007;82(9):1091–102. 10.4065/82.9.1091 . 17803877

[34] New York State Department of Health. Medicaid screening for syphilis among adult males living with HIV by region (unpublished data); Internal NYSDOH collaboration 2018.

[35] Hart-Malloy R, Rosenthal, M., Patterson, W., Cukrovany, J., Currenti, S., Gunn, JLK, editor Syphilis diagnosis among men who have sex with men who are living with diagnosed HIV infection: Assessing morbidity in New York State (excluding New York City). STD Prevention Conference; 2018; Washington DC.

[36] Centers for Disease Control and Prevention. Monitoring selected national HIV prevention and care objectives by using HIV surveillance data—United States and 6 dependent areas, 2015. HIV Surveillance Supplemental Report 2017.

[37] HIV Reporting and Partner Notification Law Article 21 Title III. Sect. 2139 (1998).

[38] New York State Codes, Rules, and Regulations; Volume A-1a (Title 10) SubChapter G- AIDS Testing, Communicable Diseases and Poisoning; Part 63- HIV/AIDS Testing, Reporting and Confidentiality of HIV-Related Information; Section 63.4: Filing of Reports, (05/17/2017, 2017).

[39] Centers for Disease Control and Prevention. National Notifiable Diseases Surveillance System (NNDSS), Surveillance Case Definitions, Syphilis (Treponema pallidum) [cited 2018]. Available from: https://wwwn.cdc.gov/nndss/conditions/syphilis/.

[40] United States Census Bureau. American Community Survey, 2017 American Community Survey 5-Year Estimate [5/14/2018]. Available from: http://factfinder.census.gov/.

[41] Division of STD Prevention National Center for HIV/AIDS Viral Hepatitis STD and TB Prevention Centers for Disease Control and Prevention. Syphilis—CDC Fact Sheet (Detailed) 2017 [updated 11/30/2017; cited 2018]. Available from: https://www.cdc.gov/std/syphilis/stdfact-syphilis-detailed.htm.

[42] Cherneskie T. An Update and Review of the Diagnosis and Management of Syphilis. Region II STD/HIV Prevention Training Center, New York City Department of Health and Mental Hygiene; New York, NY: 2006.

[43] Callegari FM, Pinto-Neto LF, Medeiros CJ, Scopel CB, Page K, Miranda AE. Syphilis and HIV co-infection in patients who attend an AIDS outpatient clinic in Vitoria, Brazil. AIDS Behav. 2014;18 Suppl 1:S104–9. 10.1007/s10461-013-0533-x 23732958 PMC3818508

[44] Tesoriero JM, Johnson BL, Hart-Malloy R, Cukrovany JL, Moncur BL, Bogucki KM, et al Improving Retention in HIV Care Through New York's Expanded Partner Services Data-to-Care Pilot. Journal of Public Health Management and Practice. 2017;23(3):255–63. 10.1097/PHH.0000000000000483 00124784-201705000-00004. 27902561 PMC5381495

[45] Quinn TC, Wawer MJ, Sewankambo N, Serwadda D, Li C, Wabwire-Mangen F, et al Viral load and heterosexual transmission of human immunodeficiency virus type 1. Rakai Project Study Group. N Engl J Med. 2000;342(13):921–9. 10.1056/NEJM200003303421303 . 10738050

[46] Marks G, Patel U, Stirratt MJ, Mugavero MJ, Mathews WC, Giordano TP, et al Single Viral Load Measurements Overestimate Stable Viral Suppression Among HIV Patients in Care: Clinical and Public Health Implications. J Acquir Immune Defic Syndr. 2016;73(2):205–12. 10.1097/QAI.0000000000001036 27105049 PMC5964607

[47] Stirratt MJ, Marks G, O'Daniels C, Cachay ER, Sullivan M, Mugavero MJ, et al Characterising HIV transmission risk among US patients with HIV in care: a cross-sectional study of sexual risk behaviour among individuals with viral load above 1500 copies/mL. Sex Transm Infect. 2018;94(3):206–11. 10.1136/sextrans-2017-053178 29097417 PMC5958893

[48] New York State Department of Health. Partner Services Data to Care Report, New York State (excluding New York City) 2015 [Agency Report]. 2017. Available from: https://www.health.ny.gov/diseases/aids/general/statistics/docs/partner_services.pdf.

