# Supplementary material for: Syphilis among adult males with a history of male-to-male sexual contact living with diagnosed HIV in New York State (excluding New York City): The challenge of intersecting epidemics
Source: PLoS One. 2019 Dec 18;14(12):e0226614. doi: 10.1371/journal.pone.0226614 (PMC6919591; doi:10.1371/journal.pone.0226614)
Supplement: S1 File — (Table A) Supplemental Descriptive Analysis for Cohort of Adult Males with a History of Male-to-Male Sexual Contact Living with Diagnosed HIV, and Syphilis Diagnosis Status: New York State Excluding New York City, 2013–2016. (Table B) Supplemental Analysis of Syphilis Diagnosis on the Same Day as HIV Care Among MSM over the Age of 18 Living With HIV by Demographic Factors, New York State Excluding New York City, 2016. (DOCX) [file pone.0226614.s001.docx]

| **Table A. Supplemental Descriptive Analysis for Cohort of Adult Males with a History of Male-to-Male Sexual Contact Living with Diagnosed HIV, and Syphilis Diagnosis Status: New York State Excluding New York City, 2013-2016** | | | | | | | | |
| --- | --- | --- | --- | --- | --- | --- | --- | --- |
|  | **Total** | | **Syphilis Diagnosis** | | **No Syphilis** | | Unadjusted Risk Ratio and 95% CL^1^ | Adjusted Risk Ratio and 95% CL^1^ |
|  | (n) | col % | (n) | col % | (n) | col % |  |  |
| **Total** | 7,512 | - | 320 | - | 7,192 | - | - | - |
| **Race/Ethnicity** |  |  |  |  |  |  |  |  |
| Non-Hispanic White | 3,755 | 50.0% | 122 | 38.1% | 3,633 | 50.5% | *Ref.* | *Ref.* |
| Non-Hispanic Black | 1,386 | 18.5% | 88 | 27.5% | 1,298 | 18.0% | **1.95 (1.49-2.55)** | **1.47 (1.12-1.93)** |
| Hispanic | 1,434 | 19.1% | 66 | 20.6% | 1,368 | 19.0% | **1.41 (1.05-1.89)** | 1.05 (0.78-1.40) |
| Other^2^ | 937 | 12.5% | 44 | 13.8% | 893 | 12.4% | **1.44 (1.03-2.02)** | 1.19 (0.85-1.66) |
| **Age Group^3^** |  |  |  |  |  |  |  |  |
| 18 - 24 | 137 | 1.8% | 17 | 5.3% | 120 | 1.7% | **5.74 (3.55-9.30)** | **3.06 (1.81-5.18)** |
| 25 - 34 | 956 | 12.7% | 110 | 34.4% | 846 | 11.8% | **5.33 (4.13-6.87)** | **3.04 (2.25-4.09)** |
| 35 - 44 | 1,281 | 17.1% | 82 | 25.6% | 1,199 | 16.7% | **2.96 (2.24-3.92)** | **2.15 (1.60-2.87)** |
| 45+ | 5,138 | 68.4% | 111 | 34.7% | 5,027 | 69.9% | *Ref.* | *Ref.* |
| **Viral Load <1500 c/mL** |  |  |  |  |  |  |  |  |
| Always^4^ | 3,867 | 51.5% | 169 | 52.8% | 3,698 | 51.4% | **2.98 (1.79-4.95)** | **4.09 (2.46-6.81)** |
| Sometimes^5^ | 2,554 | 34.0% | 135 | 42.2% | 2,419 | 33.6% | **3.60 (2.15-6.02)** | **3.60 (2.16-6.00)** |
| Never^6^ | 1,091 | 14.5% | 16 | 5.0% | 1,075 | 14.9% | *Ref.* | *Ref.* |
| **HIV Care Status** |  |  |  |  |  |  |  |  |
| Always^7^ | 4,982 | 66.3% | 225 | 70.3% | 4,757 | 66.1% | **5.18 (2.57-10.40)** | - |
| Sometimes^8^ | 1,611 | 21.4% | 87 | 27.2% | 1,524 | 21.2% | **6.20 (3.02-12.70)** | - |
| Never^9^ | 919 | 12.2% | 8 | 2.5% | 911 | 12.7% | *Ref.* | - |
| **Duration of HIV Positive Status** |  |  |  |  |  |  |  |  |
| 1-3 Years | 1,098 | 14.6% | 100 | 31.3% | 998 | 13.9% | **7.83 (5.38-11.4)** | **3.77 (2.47-5.76)** |
| 4-6 Years | 1,033 | 13.8% | 81 | 25.3% | 952 | 13.2% | **6.74 (1.48-9.92)** | **3.90 (2.56-5.93)** |
| 7-13 years | 2,287 | 30.4% | 103 | 32.2% | 2,184 | 30.4% | **3.87 (2.66-5.64)** | **2.88 (1.96-4.25)** |
| Greater than 13 years | 3,094 | 41.2% | 36 | 11.3% | 3,058 | 42.5% | *Ref.* | *Ref.* |
| **Zip Code Percent Below Poverty** |  |  |  |  |  |  |  |  |
| 0% - 5.7% | 1,100 | 14.6% | 56 | 17.5% | 1,044 | 14.5% | 1.17 (0.86-1.59) | - |
| 5.7% - 10.2% | 1,824 | 24.3% | 69 | 21.6% | 1,755 | 24.4% | 0.86 (0.63-1.17) | - |
| 10.2% - 16.4% | 1,445 | 19.2% | 62 | 19.4% | 1,383 | 19.2% | 0.96 (0.68-1.34) | - |
| 16.4% and higher | 2,894 | 38.5% | 129 | 40.3% | 2,765 | 38.4% | *Ref.* | - |
| Missing | 249 | 3.3% | 4 | 1.3% | 245 | 3.4% | - | - |
| **Zip Code Unemployment Rate** |  |  |  |  |  |  |  |  |
| Below 4.4% | 763 | 10.2% | 35 | 10.9% | 728 | 10.1% | 0.95 (0.54-1.67) | - |
| Between 4.4% and 6.5% | 2,355 | 31.3% | 82 | 25.6% | 2,273 | 31.6% | 0.93 (0.65-1.32) | - |
| Between 6.5% and 9.2% | 2,481 | 33.0% | 128 | 40.0% | 2,353 | 32.7% | 1.09 (0.84-1.42) | - |
| Higher than 9.2% | 1,663 | 22.1% | 70 | 21.9% | 1,593 | 22.1% | *Ref.* | - |
| Missing | 250 | 3.3% | 5 | 1.6% | 245 | 3.4% | - | - |
| **Zip Code Percent Uninsured** |  |  |  |  |  |  |  |  |
| Below 4.0% | 1,006 | 13.4% | 48 | 15.0% | 958 | 13.3% | 1.05 (0.73-1.49) | - |
| Between 4.0% and 6.3% | 1,893 | 25.2% | 62 | 19.4% | 1,831 | 25.5% | 0.72 (0.52-1.01) | - |
| Between 6.3% and 9.3% | 2,458 | 32.7% | 118 | 36.9% | 2,340 | 32.5% | 1.04 (0.77-1.39) | - |
| Above 9.3% | 1,907 | 25.4% | 88 | 27.5% | 1,819 | 25.3% | *Ref.* | - |
| Missing | 248 | 3.3% | 4 | 1.3% | 244 | 3.4% | - | - |
| 1. Bolded results indicate p-value<0.05 2. Other race includes Asian/Pacific Islander, Native American, Multi-race, and unknown 3. Persons age as of Jan. 1, 2014 4. Always: from January 1, 2013 to December 31, 2016, all viral loads must be <1,500 viral c/mL and HIV viral load laboratory test collection dates needed to be < 13 months apart 5. Sometimes includes one of the following two conditions: 1) all test results were <1,500c/mL but there were gaps of HIV care > 13 months between tests or 2) or any test was > 1,500 c/mL, but there was at least one test < 1,500 c/mLl. 6. Never: zero tests <1,500 c/mL or no viral load test dates reported to the NYSDOH 7. Always: presence of at least one HIV-related laboratory test per year of follow-up (i.e. HIV-related laboratory testing collection dates < 13 months apart) 8. Sometimes: at least one HIV-related laboratory test during follow-up (i.e. HIV-related laboratory testing collection date(s) >13 months) 9. Never: no evidence of an HIV-related laboratory reported to the NYSDOH within study period | | | | | | | | |
|  | | | | | | | | |
|  | | | | | | | | |

| **Table B. Supplemental Analysis of Syphilis Diagnosis on the Same Day as HIV Care Among MSM over the Age of 18 Living With HIV by Demographic Factors, New York State Excluding New York City, 2016** | | | | | | | |
| --- | --- | --- | --- | --- | --- | --- | --- |
|  | **Total^1^** | | **Same Day Diagnosis^2^** | | | | **Risk Ratio and 95% CL^3^** |
|  |  | | Yes | | No | |  |
|  | (n) | col % | (n) | col % | (n) | col % |  |
| **Total** | 312 |  | 191 | - | 121 | - |  |
| **Syphilis Disease Stage** |  |  |  |  |  |  |  |
| Primary Syphilis | 51 | 16.3% | 26 | 13.6% | 25 | 20.7% | *Ref.* |
| Secondary Syphilis | 107 | 34.3% | 54 | 28.3% | 53 | 43.8% | 0.99 (0.71-1.37) |
| Early Latent Syphilis | 154 | 49.4% | 111 | 58.1% | 43 | 35.5% | **1.41 (1.06-1.88)** |
| **Race/Ethnicity^4^** |  |  |  |  |  |  |  |
| Non-Hispanic White | 120 | 38.5% | 80 | 41.9% | 40 | 33.1% | *Ref.* |
| Non-Hispanic Black | 84 | 26.9% | 45 | 23.6% | 39 | 32.2% | 0.80 (0.63-1.02) |
| Hispanic | 64 | 20.5% | 40 | 20.9% | 24 | 19.8% | 0.94 (0.75-1.18) |
| Other | 44 | 14.1% | 26 | 13.6% | 18 | 14.9% | 0.89 (0.67-1.17) |
| **Age Group^5^** |  |  |  |  |  |  |  |
| 18 - 24 | 17 | 5.4% | 11 | 5.8% | 6 | 5.0% | 0.99 (0.68-1.45) |
| 25 - 34 | 106 | 34.0% | 59 | 30.9% | 47 | 38.8% | 0.85 (0.69-1.06) |
| 35 - 44 | 80 | 25.6% | 50 | 26.2% | 30 | 24.8% | 0.96 (0.77-1.19) |
| 45 - 54 | 109 | 34.9% | 71 | 37.2% | 38 | 31.4% | *Ref.* |
| **Viral Load Always <1500 c/mL** |  |  |  |  |  |  |  |
| Yes | 169 | 54.2% | 107 | 56.0% | 62 | 51.2% | 1.08 (0.90-1.29) |
| No | 143 | 45.8% | 84 | 44.0% | 59 | 48.8% | *Ref.* |
| **HIV Care Status** |  |  |  |  |  |  |  |
| Always^6^ | 225 | 72.1% | 145 | 75.9% | 80 | 66.1% | 1.22 (0.98-1.52) |
| Sometimes in Care^7^ | 87 | 27.9% | 46 | 24.1% | 41 | 33.9% | *Ref.* |
| Never in Care | - | - | - | - | - | - | *excluded* |
| **Duration of HIV Positive Status** |  |  |  |  |  |  |  |
| 1-3 Years | 98 | 31.4% | 61 | 31.9% | 37 | 30.6% | 0.83 (0.65-1.06) |
| 4-6 Years | 79 | 25.3% | 42 | 22.0% | 37 | 30.6% | **0.71 (0.54-0.94)** |
| 7-13 years | 99 | 31.7% | 61 | 31.9% | 38 | 31.4% | 0.82 (0.64-1.05) |
| Greater than 13 years | 36 | 11.5% | 27 | 14.1% | 9 | 7.4% | *Ref.* |
| **Missed Opportunity^8^** |  |  |  |  |  |  |  |
| Yes | 145 | 46.5% | 83 | 43.5% | 62 | 51.2% | *Ref.* |
| No | 167 | 53.5% | 108 | 56.5% | 59 | 48.8% | 1.13 (0.94-1.35) |

1. Excludes those who had no HIV-related laboratory test collection dates (N=8)
2. Same day syphilis diagnosis: syphilis diagnosed date matches an HIV care date
3. Bolded results indicate *p*-value<0.05
4. Other race includes Asian/Pacific Islander, Native American, Multi-race, and unknown
5. Persons age as of January 1, 2014
6. Always: presence of at least one HIV-related laboratory test per year of follow-up (i.e. HIV-related laboratory testing collection dates <13 months apart)
7. Sometimes: at least one HIV-related laboratory test during follow-up (i.e. HIV-related laboratory testing collection date(s) >13 months)
8. Missed opportunity: an HIV care date within 3 months prior to a secondary syphilis diagnosis, or 6 months prior to an early latent syphilis diagnosis
